# Mapping of the Lassa virus LAMP1 binding site reveals unique determinants not shared by other old world arenaviruses

**DOI:** 10.1371/journal.ppat.1006337

**Published:** 2017-04-27

**Authors:** Hadar Israeli, Hadas Cohen-Dvashi, Anastasiya Shulman, Amir Shimon, Ron Diskin

**Affiliations:** Department of Structural Biology, Weizmann Institute of Science, Rehovot, Israel; Harvard Medical School, UNITED STATES

## Abstract

Cell entry of many enveloped viruses occurs by engagement with cellular receptors, followed by internalization into endocytic compartments and pH-induced membrane fusion. A previously unnoticed step of receptor switching was found to be critical during cell entry of two devastating human pathogens: Ebola and Lassa viruses. Our recent studies revealed the functional role of receptor switching to LAMP1 for triggering membrane fusion by Lassa virus and showed the involvement of conserved histidines in this switching, suggesting that other viruses from this family may also switch to LAMP1. However, when we investigated viruses that are genetically close to Lassa virus, we discovered that they cannot bind LAMP1. A crystal structure of the receptor-binding module from Morogoro virus revealed structural differences that allowed mapping of the LAMP1 binding site to a unique set of Lassa residues not shared by other viruses in its family, illustrating a key difference in the cell-entry mechanism of Lassa virus that may contribute to its pathogenicity.

## Author summary

To infect, enveloped viruses need to fuse their membrane with the host membrane. Fusion is mediated by special glycoprotein machineries that must be triggered only at the right time and at the right place. A major cue that viruses utilize for triggering is acidic pH. Until recently, such pH-induced triggering was assumed to be the only mechanism used by the *Arenaviridae* family. However, Lassa virus, a notorious pathogenic member of this family, was shown to use the binding to an intracellular receptor named LAMP1 to potentiate its pH-induced triggering. This two-step mechanism was a surprising finding that raised critical questions regarding the cell-entry mechanisms of other viruses from this family. Here we used a structure-guided approach to investigate whether other *Arenaviridae* utilize LAMP1 for cell entry. We mapped the LAMP1 binding site on the Lassa-derived glycoprotein and confirmed its identity using grafting experiments. This mapping revealed the unique sequence signature needed for LAMP1 binding. Sequence analysis suggests that no other members of the *Arenaviridae* bind LAMP1.

## Introduction

Receptor switching is a newly discovered event in the cell entry process of Lassa virus (LASV) [1], a zoonotic, enveloped, negative-strand RNA virus that belongs to the *Arenaviridae* family [2]. LASV is a devastating pathogen that causes severe hemorrhagic fevers with significant mortality in West Africa [3]. LASV locates its host cells by binding to its primary cellular receptor, α-dystroglycan [4, 5]. Then, through a process of macropinocytosis [6, 7], LASV is internalized and reaches a late endosomal compartment. In this acidifying environment, LASV changes its binding specificity and engages LAMP1, a ubiquitous protein of lysosomes and late endosomes [1]. A requirement for receptor switching has also been identified for Ebola virus, which binds to the Neimann-Pick C1 protein in the lysosome to infect cells [8, 9]. Receptor switching is thus an emerging theme for viral entry that may be relevant for other viruses as well.

LASV has a surface-displayed class-I trimeric glycoprotein spike complex that mediates receptor recognition and fusion of the viral and host-cell membranes at acidic pH [10, 11]. The spike complex consists of three copies of a single polypeptide chain that is cleaved twice to give a structured signal peptide, a receptor-binding module (GP1), and a trans-membrane module (GP2) [12]. We previously showed that a triad of histidines on GP1 is important for binding LAMP1 [13], and we further demonstrated that the binding of LAMP1 triggers the spike of LASV to catalyze membrane fusion by potentiating its response to pH [14]. Upon protonation in a weak acidic environment, the positively charged histidine triad functions to inhibit pre-mature triggering of the spike, an inhibition that LAMP1 overrides [14].

LASV is classified as an ‘Old World’ (OW) mammarenavirus [15]. The histidine triad is fully conserved among OW mammarenaviruses and thus may have a similar function in these viruses as well. A critical question is whether other OW mammarenaviruses are activated by LAMP1 binding during cell entry. Here we investigate this possibility and show that representative OW mammarenaviruses do not interact with LAMP1. We present a crystal structure of the GP1 domain from the Morogoro (MORV) OW mammarenavirus [16], which does not interact with LAMP1, and conduct a comparative structural analysis between GP1 of MORV and LASV (GP1_MORV_ and GP1_LASV_, respectively) to identify structural differences related to the ability to bind LAMP1. Structure-guided mutagenesis assisted mapping of the LAMP1 binding site on GP1_LASV_, which was corroborated by grafting it onto GP1_MORV_. The binding site is located on the apex of the trimeric spike complex, suggesting critical attributes for the activation mechanism of the LASV spike complex by LAMP1. Moreover, the interaction surface includes a variable region of LASV that significantly differs from other OW mammarenaviruses. Thus, we conclude that switching to LAMP1 is unique for LASV.

## Results

### MORV and LCMV cannot bind human LAMP1

**Fig 1:**
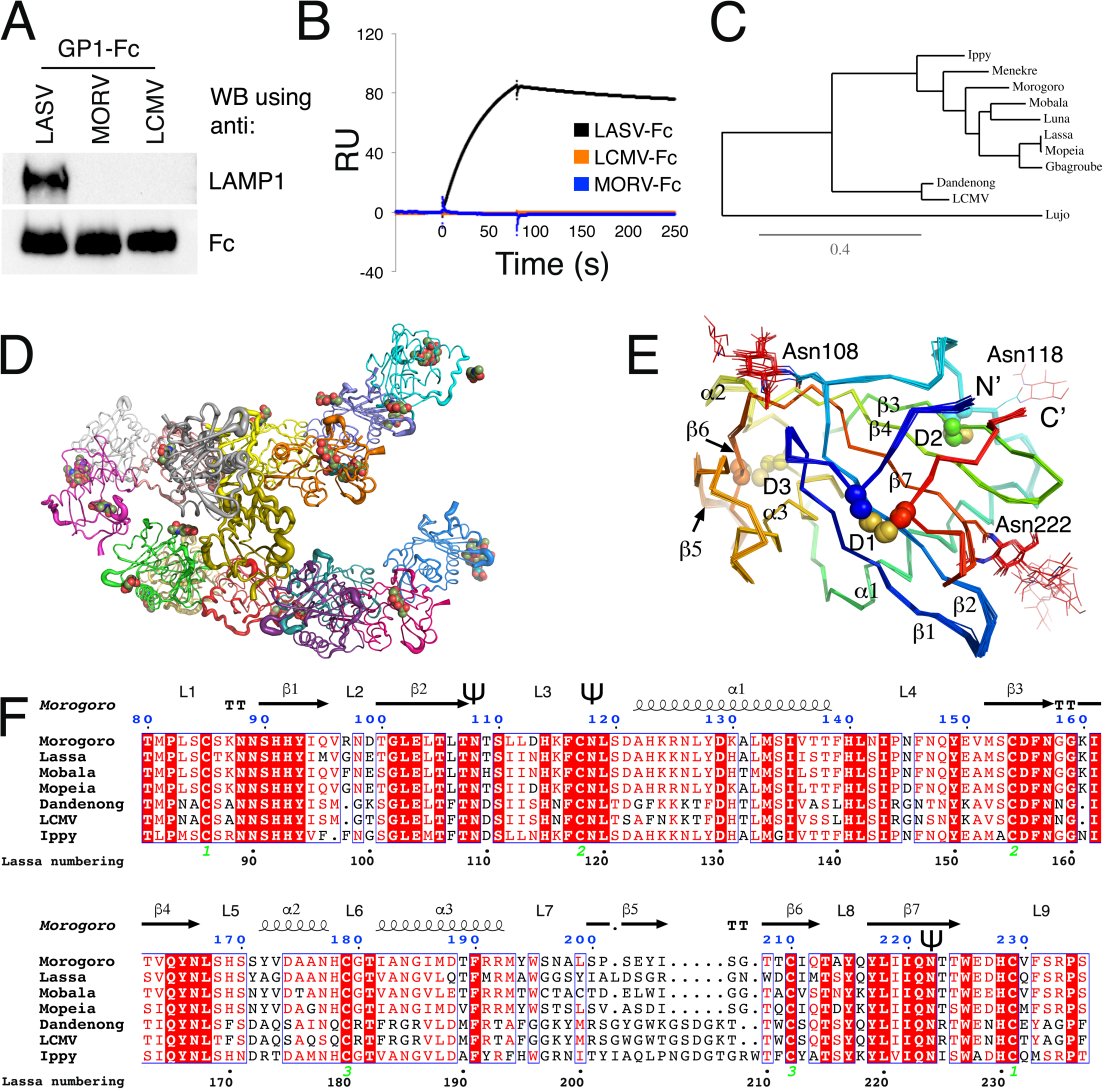
GP1_LCMV_ and GP1_MORV_ do not interact with LAMP, although GP1_MORV_ adopts the same global fold as GP1_LASV_. (A) Pull-down assay by the indicated GP1-Fc fusion proteins. The presence of LAMP1 is detected by anti-LAMP1 antibody, and Fc levels are shown using anti-Fc for load control. The pull-down assay was independently repeated three times, and a representative image is shown. (B) SPR analysis of the indicated GP1-Fc analytes. Each analyte was injected at 500 nM over immobilized distal domain of LAMP1. (C) A phylogenetic tree based on the sequences of the GPCs from the indicated OW mammarenaviruses. The scale bar represents a substitution rate of 0.4 per site. (D) Graphical representation of the 16 chains, each colored differently, that make the asymmetric unit. The chains are traced as tubes with a radius proportional to B-factor. N-linked glycans are shown as spheres. (E) Cα traces of all 16 chains superimposed. N-linked glycans are shown with lines. The cysteine residues that make the three disulfide bonds (marked with a ‘D’) are shown with spheres and are labeled, and secondary structure elements are numbered. (F) Multiple sequence alignment of GP1s from the indicated OW mammarenaviruses showing the secondary structure elements as observed in the crystal structure of GP1_MORV_. The numbering of the amino acids is based on the sequence of GPC_MORV_. Fully conserved residues are highlighted with red background, and partially conserved residues are shown in red. Numbers below the sequences mark the locations and connectivity of the disulfide bridges. The symbol ‘Ψ’ marks the locations of N-linked glycans seen in the crystal structure. We used ESPript [21] (http://espript.ibcp.fr) for generating this graphical representation.

To test whether other OW mammarenaviruses can interact with LAMP1, we produced the GP1 domain from lymphocytic choriomeningitis virus (LCMV) fused to an Fc portion of an antibody (GP1_LCMV_-Fc) and performed a pull-down assay using a total cell lysate of HEK293T cells, side by side with GP1_LASV_-Fc (Fig 1A). Unlike GP1_LASV_-Fc, GP1_LCMV_-Fc did not pull down endogenous LAMP1 (Fig 1A). We further used surface plasmon resonance (SPR) to test for potential weak interactions with the recombinant distal domain of LAMP1 (Fig 1B), which is sufficient for LASV to bind [1]. In contrast to GP1_LASV_-Fc, GP1_LCMV_-Fc was inert towards LAMP1 (Fig 1B). This observation agrees with previous indications that LAMP1 is not required for infection by LCMV [1]. A phylogenetic analysis based on the GPC sequences from representative OW mammarenaviruses shows that LCMV and LASV segregate to two different linages (Fig 1C). We thus asked whether an OW mammarenavirus that is genetically closer to LASV would be able to bind LAMP1. MORV is one of the closest OW mammarenaviruses to LASV. We therefore produced a GP1_MORV_-Fc protein and tested its ability to pull down endogenous LAMP1 (Fig 1A) or to interact with recombinant LAMP1 using SPR analysis (Fig 1B). Similarly to GP1_LCMV_-Fc, GP1_MORV_-Fc did not pull down LAMP1 from HEK293T cells, nor did it interact with recombinant LAMP1 immobilized on a sensor chip (Fig 1B).

### GP1_MORV_ assumes a global “primed” conformation similarly to GP1_LASV_

Because GP1_MORV_ did not bind LAMP1, we first sought to determine whether GP1_MORV_ assumes a similar conformation to that previously observed for the LAMP1 binding-competent GP1_LASV_. In this regard, it was recently shown that the prefusion-conformation of GPC_LCMV_ [17] greatly differs from the conformation of isolated GP1_LASV_ [13] and inability to adopt a similar conformation to GP1_LASV_ could thus account for the inability to bind LAMP1. To investigate that, we crystallized and solved the structure of GP1_MORV_ to 2.6 Å resolution (PDB: 5NFF). We obtained crystals of GP1_MORV_ (residues 73 to 235) at pH 4.0. Crystals belonged to a trigonal (*P*3_2_) space group (Table 1). We found a molecular replacement solution using the coordinates of GP1_LASV_ (PDB: 4ZJF) as a search model. The asymmetric unit contained a total of 16 copies of GP1_MORV_ (Fig 1D), mostly differing in the extent of electron density observed for their N-linked glycans, which in some cases were stabilized by a nearby protein molecule. Electron density allowed us to model N-linked glycans at position 108 for 11 chains and at position 222 for 7 chains. Chain B was modeled with an N-linked glycan at position 118 as well. Due to crystal packing, chains differed in their average thermal factors (Fig 1D). The model was refined utilizing restraints between non-crystallographic symmetry-related molecules. We utilized simulated annealing and omission of selected regions during refinement to eliminate model bias. The final model of GP1_MORV_ consists of residues 80–235 for all 16 chains and has R_work_/R_free_ values of 17.4% and 20.7%, respectively (Table 1).

**Table 1:** Data collection and refinement statistics.

Data collection^#^	
Wavelength	0.9194
Space group	*P* 3_2_
**Cell dimensions**	
*a*, *b*, *c* (Å)	127.8, 127.8, 251.7
α, β, γ (°)	90, 90, 120
Resolution (Å)	46.19–2.61
R_meas_ (%)	14.9 (96.3)
*CC*_*1/2*_	98.7 (35)
*I/σI*	9.09 (1.58)
Completeness (%)	98.9 (92.2)
Multiplicity	4.0 (3.8)
Reflections	554,160
Unique reflections	138,414
**Refinement**	
Resolution (Å)	46.19–2.61
No. Reflections	138,413
R_work_/R_free_^a^	17.4/20.7
No. of atoms	1246
Protein	19,936
Ligand/ion	455
**B factors**	
Protein	55.42
Ligand	68.52
**Ramachandran**	
Favored (%)	98
Allowed (%)	2.5
Outlier (%)	0
**Root mean square deviations**	
Bond length (Å)	0.01
Bond angles	1.18

# Values in parentheses correspond to the high-resolution shell (2.7–2.61).

^a^ The size of the test set was 5.04% of all data.

**Fig 2:**
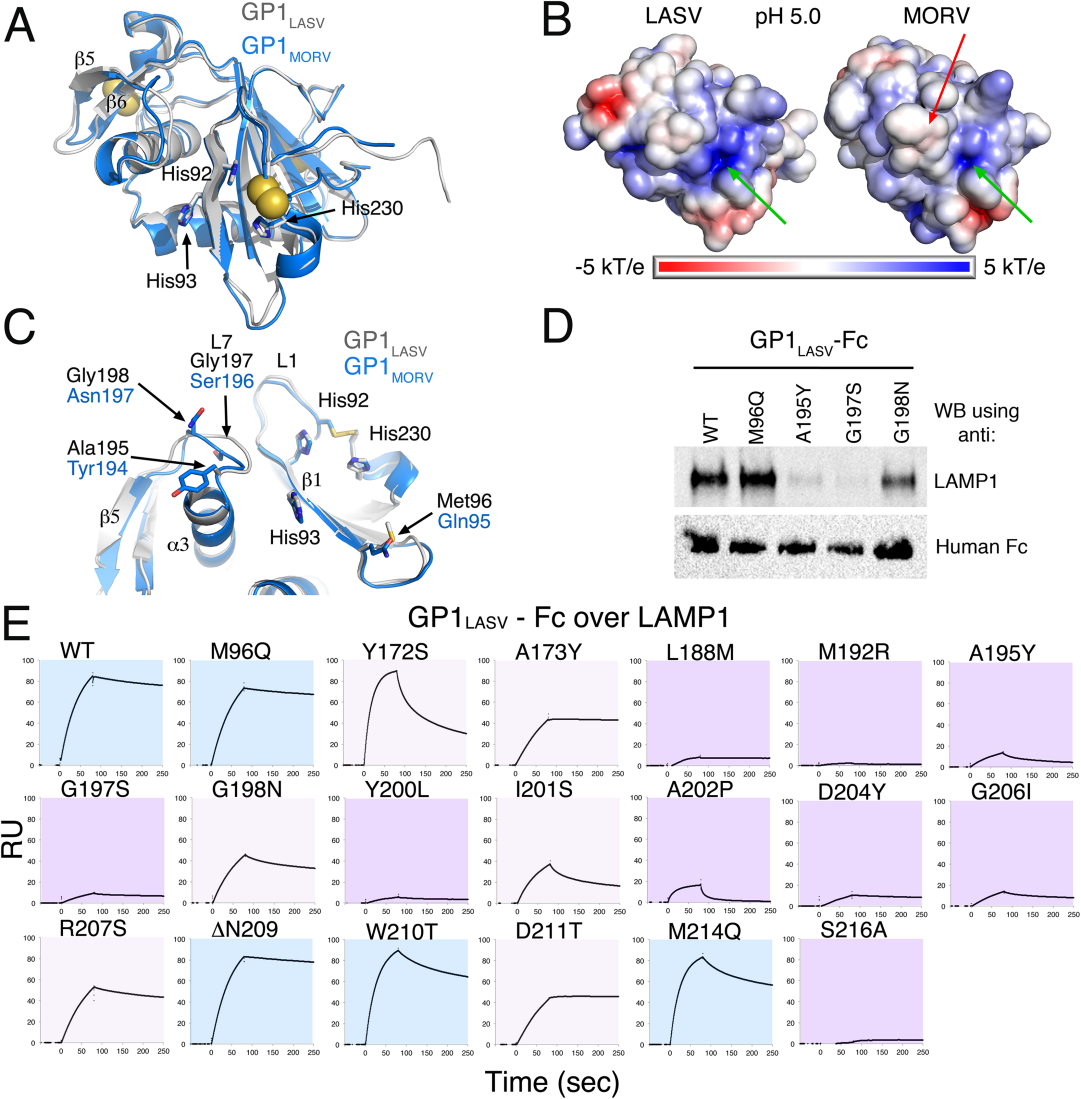
Structural comparison between GP1_MORV_ and GP1_LASV_ and the vicinity of the histidine triad. (A) Superimposition of GP1_MORV_ (blue) and GP1_LASV_ (grey) (PDB: 4ZJF). The histidine triad is indicated as well as β5 & β6. Disulfides are shown with spheres for the sulfur atoms (B) The solvent accessible surfaces of GP1_MORV_ and GP1_LASV_ are presented on the right and left sides, respectively. The proteins are positioned as in panel ‘A’, and the histidine triads are indicated with green arrows. A red arrow marks bulky residues on GP1_MORV_. The surfaces are colored according to the electrostatic potential in the range of ± 5 kT/e, as calculated by APBS tools at pH 5.0. (C) A close up view of the vicinity of the histidine triad in a superimposition of GP1_MORV_ (blue) and GP1_LASV_ (grey). Non-conserved sites in this region are indicated. (D) Pull-down experiment of LAMP1 by the indicated GP1_LASV_-Fc mutants. A representative image of three independent repeats. (E). SPR analyses of GP1_LASV_ point mutations. The various mutants in GP1_LASV_ were injected as analytes at 450 nM over immobilized distal domain of LAMP1. WT GP1_LASV_ was injected multiple times at the beginning, middle and end of the injection series to monitor the consistency of the immobilized LAMP1, producing indistinguishable sensograms (only a representative curve is shown). The binding curves were manually inspected and binned into three groups: showing no or very little effect on binding (light-blue background), moderately reduced binding (pink background), and strongly diminished binding (purple background).

GP1_MORV_ has a central β–sheet made of five β-strands, flanked by the domain termini on one side and helices and loops on the other (Fig 1E & 1F). The global structure of GP1_MORV_ is similar to the previously determined structure of GP1_LASV_ [13] (Fig 2A). The histidine triad in GP1_MORV_ (His91, His92, and His228 according to GPC_MORV_ numbering) adopts the same conformation as the triad in GP1_LASV_ (Fig 2A). GPC_MORV_ has a β-hairpin made of two β-strands (β5 & β6) stabilized by a disulfide bridge (D3) against α3 (Fig 1E). This β-hairpin is divergent in sequence (Fig 1F) and was previously termed the variable region of GP1_LASV_ [13]. Comparing the solvent accessible surfaces and the calculated electrostatic potentials of the two proteins at pH 5.0 reveals some differences both in the charge distribution and in the surface geometry (Fig 2B). GP1_LASV_ has a strong positive charge in the vicinity of the triad, whereas this charge on the GP1_MORV_ surface seems milder (Fig 2B). On this side of the protein, GP1_MORV_ lacks one negatively charged patch compared to GP1_LASV_ and has a slightly more bulky surface near the histidine triad (Fig 2B). Overall, the structure of GP1_MORV_ does not reveal major global structural differences that could account to the inability to bind LAMP1. However, the structural similarity together with a 70% amino-acid identity between GP1_LASV_ and GP1_MORV_ provides now a unique opportunity to elucidate the structural determinants of GP1_LASV_ that are required for LAMP1 binding or of GP1_MORV_ that preclude LAMP1 binding.

### Mapping the LAMP1 binding site on GP1_LASV_

Previously we showed that mutations of histidines in the GP1_LASV_ triad to tyrosines abrogated LAMP1 binding [13] and that LAMP1 requires a positively charged His230 for binding [14]. To map other regions that contribute to the LAMP1 binding site on GP1_LASV_, we compared the vicinities of the histidine triads of GP1_LASV_ and GP1_MORV_ (Fig 2C) and found several compositional as well as conformational differences between the two proteins. One is Met96 at the carboxy terminus of LASV strand β1, which corresponds to Gln95 in MORV. Other differences are in a loop connecting helix α3 with strand β5 (Fig 2C), designated L7 (Fig 1F). These include the small residues Ala195_LASV_, Gly197_LASV_ and Gly198_LASV_, which are replaced by the more bulky Tyr194_MORV_, Ser196_MORV_ and Asn197_MORV_, respectively (Fig 2C). To evaluate whether these variations affect LAMP1 binding, we mutated the residues in GP1_LASV_-Fc to their corresponding amino acids from GP1_MORV_ and tested the ability of the mutated GP1_LASV_-Fc to pull down endogenous LAMP1 (Fig 2D) and to bind recombinant LAMP1 using SPR (Fig 2E). Whereas the M96Q mutation did not change the ability to bind LAMP1, mutations of the alanine and the first glycine residue in L7 almost completely abolished binding. Mutating Gly198_LASV_, at the end of L7, only slightly affected LAMP1 binding compared to WT GP1_LASV_ (Fig 2D). Thus, the composition of the residues that makes L7 loop is critical for LAMP1 binding.

**Fig 3:**
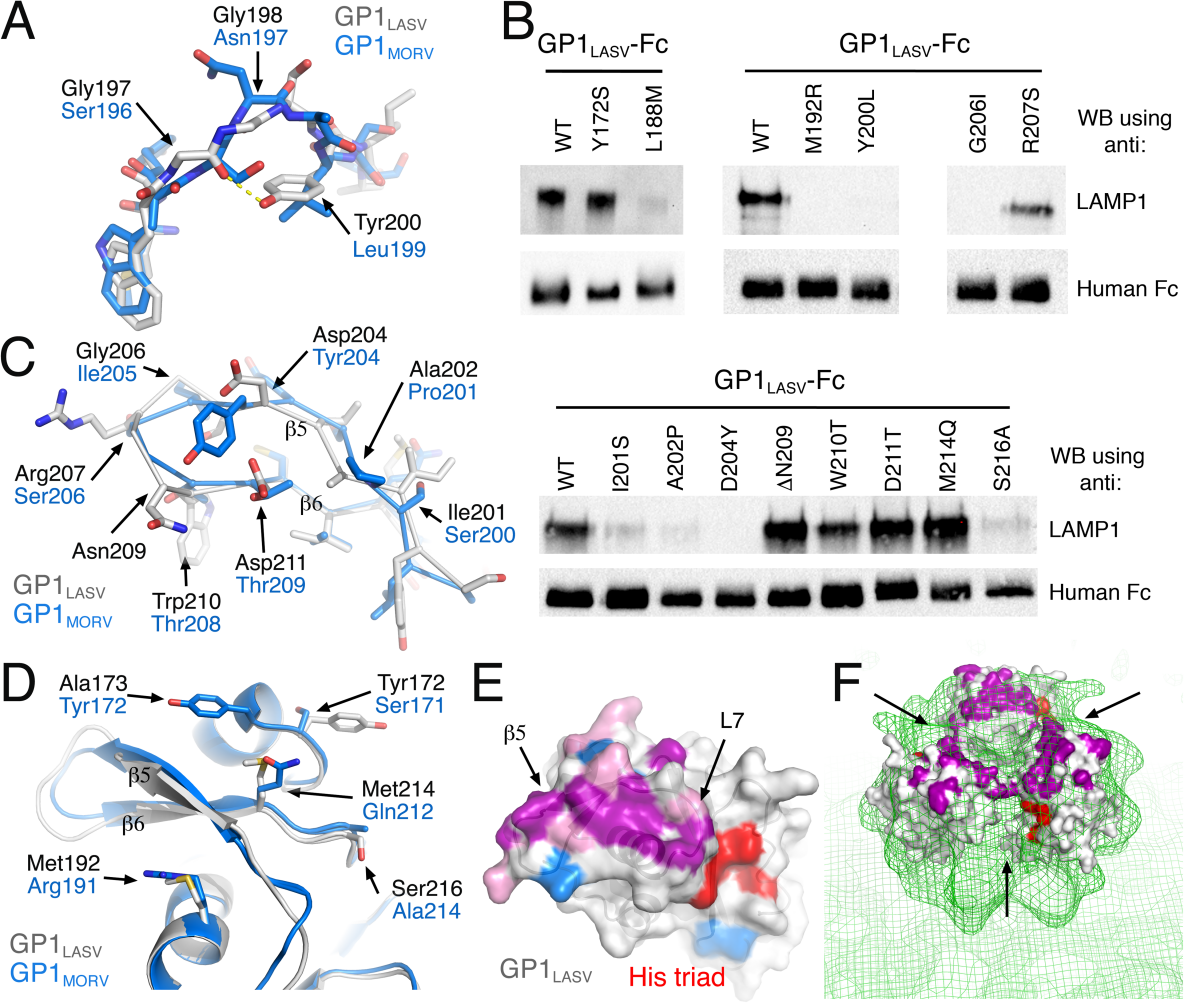
Mapping of the LAMP1 binding site on GP1_LASV_. (A) Structural comparison of the loop preceding β5. A yellow dashed line marks an apparent hydrogen bond. (B) Representative images of LAMP1 pull-down experiments by the indicated GP1_LASV_-Fc mutants, each one selected from three independent repeats. The panel is composed of three intact membranes as designated by the horizontal lines. (C) Superimposition of the β–hairpins of GP1_LASV_ and GP1_MORV_ presented as Cα traces and side-chains only. (D) Ribbon representation of the β–hairpins and their close vicinity in GP1_MORV_ and GP1_LASV_. (E) Surface representation of GP1_LASV_. Residues that were mutated and critically affected, weakly affected, or had no affect on LAMP1 binding are colored purple, pink, and blue, respectively, using the same color scheme used in Fig 2E. The histidine triad is red. (F) The crystal structure of GP1_LASV_ (white surface) docked into the EM density of the trimeric spike complex of LASV at pH 5.0 (EMDB: 3292), shown as green mesh at 1.5 σ. The histidine triad is red and all residues identified as important for LAMP1 binding are purple. The crevices between the 3 GP1 subunits are marked with black arrows.

A closer look at L7 reveals that in addition to its side-chain composition, its main-chain conformation also differs between GP1_LASV_ and GP1_MORV_ (Fig 3A). In GP1_LASV_, the loop conformation is stabilized by a hydrogen bond between the side-chain hydroxyl of Tyr200_LASV_ and the main-chain carbonyl of Gly197_LASV_ (Fig 3A). In GP1_MORV_ however, Leu199 replaces this tyrosine and cannot form a similar hydrogen bond. This in turn frees the main chain to rotate and to adopt the observed conformation (Fig 3A). Noteworthy, such main-chain conformational differences are evident in the crystal structure of GP1_MORV_ but were not predicted using a standard homology modeling approach (S1 Fig), illustrating the advantage of having *bona fide* crystallographic information for such analyses. We established that the conformation of L7 is an important determinant for LAMP1 binding, as mutating Tyr200_LASV_ to leucine prevented binding to and pull down of LAMP1 (Figs 3B & 2E). Importantly, the side chains of both Tyr200_LASV_ and Leu199_MORV_ are buried and face the protein cores, thus not likely to directly contact LAMP1. Our structural analysis further suggested that the observed conformation of L7 in GP1_LASV_ might also depend on Leu188_LASV_, a core residue that is in a close proximity to Tyr200_LASV_ (S2 Fig). Indeed, mutating Leu188_LASV_ to methionine as found in MORV substantially affected the binding to LAMP1 (Figs 3B & 2E). Hence both the composition and the conformation of L7 loop are important for binding to LAMP1. The identification of L7 as a major element for binding suggests that the binding site extends from the histidine triad toward β5 (Fig 2C).

The β–hairpin made by β5 & β6 is the most variable region in GP1s of OW mammarenaviruses (Fig 1F). It is oriented such that β5 is highly exposed and β6 is partially buried (Fig 2A). Comparing the β–hairpins from GP1_LASV_ and GP1_MORV_ indicates differences in main-chain conformation, side chains that project to opposite directions, and divergent residue composition (Fig 3C). The β–hairpin in GP1_LASV_ is one residue longer compared to GP1_MORV_, having Asn209_LASV_ as the extra residue (Fig 3C). Ile201_LASV_, Ala202_LASV_, Asp204_LASV_, and Gly206_LASV_ are exposed residues on β5, and mutating each of them individually to the corresponding residue based on the GP1_MORV_ structure (to serine, proline, tyrosine, and isoleucine, respectively) strongly diminished the ability of GP1_LASV_-Fc to pull down endogenous LAMP1 (Fig 3B) or to bind recombinant LAMP1 (Fig 2E). Asp204_LASV_ in this regard makes the extra negatively charged patch on the surface of GP1_LASV_ that is not seen in GP1_MORV_ (Fig 2B). Mutating Arg207_LASV,_ which is located at the tip of the β–hairpin, to serine had only a mild effect on the ability to interact with LAMP1 (Figs 3B & 2E), possibly marking a boundary of the LAMP1 binding site. Likewise, deleting Asn209_LASV_, mutating Trp210_LASV_, or mutating Asp211_LASV_ from β6 to threonine had either no effect or only weak effect on LAMP1 binding (Figs 3B & 2E). Following the observation that β5 is an important determinant for LAMP1 binding, we further analyzed the vicinity of β5 to identify residues that differ between GP1_LASV_ and GP1_MORV_ and could also participate in LAMP1 binding. Met192_LASV_ from helix α3 and Ser216_LASV_ from a loop connecting strands β6 and β7 (Fig 3D) also affected the pull-down of LAMP1 upon mutagenesis to arginine and alanine, respectively (Fig 3B). On the other hand, substituting Met214_LASV_ with glutamine did not affect the pull-down of LAMP1.

The residues that were shown to be important for LAMP1 binding are mostly located at β5 and L7. Together with several residues from the close vicinity of L7 in the tertiary structure, the identified residues makes an elongated and continuous surface that stretches from the histidine triad to the tip of the β–hairpin (Fig 3E). The residues that were mutated and found to be unessential for pulling down LAMP1 are distributed around the continuous surface and mark the potential boundaries of the binding site (Fig 3E). A recent EM study provided low-resolution reconstructions of the complete spike complex of LASV in its pre-fusion conformation [11]. One of the reconstructions is of the spike complex at pH 5.0 [11], a pH that is compatible with LAMP1 binding. Examining three copies of GP1_LASV_ docked into the EM density map reveals that the identified LAMP1 binding sites clusters on the apex of the trimer around its three-fold symmetry axis (Fig 3F). Such a configuration could potentially restrict the binding of more than a single LAMP1 molecule at the same time, as we further discuss below.

### Validating the LAMP1 binding site mapping by grafting it onto GP1_MORV_

**Fig 4:**
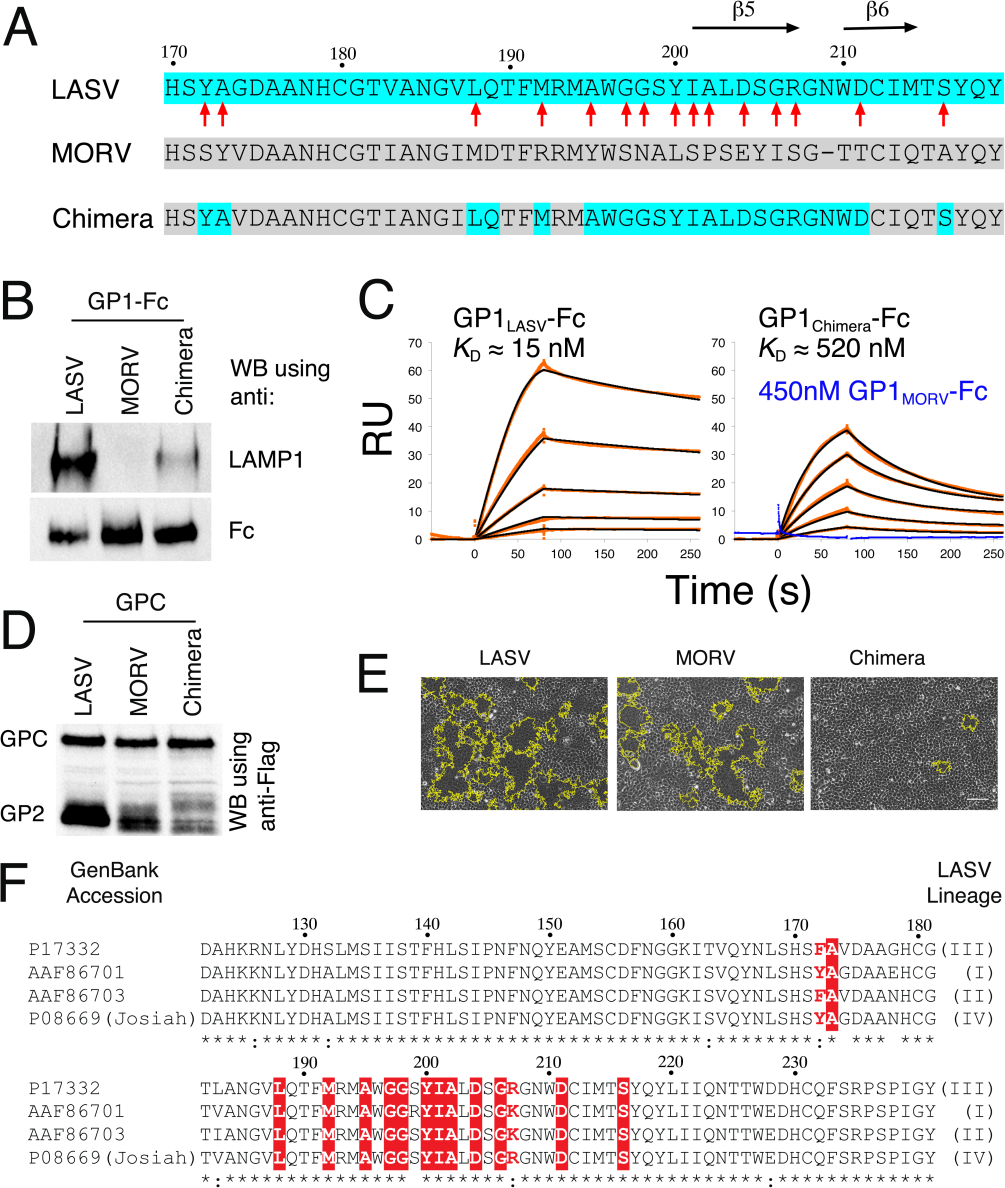
Grafting the LAMP1 binding site onto MORV. (A) Sequence alignment of residues 170–219 of GP1 (LASV numbering) from LASV, MORV, and the generated chimera. Red arrows indicate all residues identified as important for LAMP1 binding. (B) Pull-down assay of LAMP1 by the indicated GP1-Fc fusion proteins. (C) SPR analysis of the indicated GP1-Fc fusion proteins as analytes. A 2-fold dilution series of the analytes starting at 450 nM were injected over immobilized distal domain of LAMP1 (orange curves). Bivalent binding models were fitted to data (black curves), and affinity constants (*K*_D_) were calculated from the rate constants of the first binding event, when appropriate. The binding curve of 450 nM GP1_MORV_-Fc is included in the sensogram of GP1_Chimera_-Fc (blue curve) as a reference. (D) Western blot analysis using Flag-tagged GPCs. The upper molecular weight bands correspond to the full-length unprocessed GPCs. Lower molecular weight bands correspond to the cleaved GP2s. (E) Syncytia formation assays. Bright-field images of HEK293 cells showing formation of syncytia induced by the indicated GPCs. The boundaries of the syncytia are shown with yellow lines as automatically traced by ImageJ [22]. (F) Multiple sequence alignment of GP1s from LASV strains that represent the 4 major lineages. The LAMP1 binding motif is highlighted in red. Fully conserved residues indicated with a red background and chemically similar residues are shown in red font.

The functional mapping indicated that quite a few residues of GP1_MORV_ are incompatible with binding to LAMP1. In an attempt to enable GP1_MORV_ to pull down LAMP1, we created chimeric proteins by grafting critical elements from GP1_LASV_. We grafted the entire β–hairpin form GP1_LASV_ (Fig 4A), trying to ensure a correct residue composition as well as main-chain conformation. In addition, we included in these chimeric proteins Arg191_MORV_ to methionine and Ala214_MORV_ to serine substitutions as the reciprocal mutations were identified to be critical (Fig 3B & 3D) as well as Tyr172_MORV_ to alanine and Ser171_MORV_ to tyrosine mutations that also contribute to LAMP1 binding (Figs 3B & 2E). In the structure of GP1_MORV_, Arg191 interacts with and compensates the charge of Asp188 from α3. We thus further substituted Asp188_MORV_ with glutamine to avoid having an unpaired charged residue. We produced several mutated GP1 variants as Fc-fusion proteins by the addition of the various mutations in a sequential order according to the description above, performed pull down experiments for all variants, but failed to detect binding to LAMP1. Only when we included the Met187_MORV_ to leucine substitution, which corresponds to the core residue that could potentially modulate the conformation of L7 (S2 Fig) were we able to generate a chimeric GP1_MORV_ (GP1_Chimera_) capable of pulling down LAMP1 (Fig 4B). We used SPR to compare the binding of GP1_Chimera_ to LAMP1 with the binding of GP1_LASV_ (Fig 4C). Grafting the identified binding site on GP1_MORV_ resulted in a remarkable gain of LAMP1-binding function with an estimated *K*_D_ of ~520 nM (Fig 4C). Though somewhat weaker than GP1_LASV_ binding of LAMP1 *(K*_D_ of ~15 nM), this result indicates that we faithfully identified the LAMP1 binding site.

### Grafting the LAMP1 binding site onto GPC_MORV_

Next, we asked whether grafting the LAMP1 binding site onto a complete MORV spike complex would allow the formation of a functional spike and whether it could affect spike-mediated cell-entry. For this aim, we replaced a portion of the GPC_MORV_ with the GP1_Chimera_ to produce GPC_Chimera_. By introducing a Flag-tag at the C’-terminus, we were able to verify that GPC_Chimera_ expresses similarly to the WT GPCs as seen in the high molecular weight band corresponding to unprocessed GPC (Fig 4D). The processing of the spike to produce GP2, however, was less efficient and more heterogeneous than seen for GPC_MORV_ (Fig 4D). This observation suggests that GPC_Chimera_ fails to assume a fully native conformation, probably due to incompatibilities between the altered residues in GP1_Chimera_ and the native GP2_MORV_ protein. This would reduce the amount of properly processed and functional spikes displayed on GPC_Chimera_-expressing cells. Indeed, when we tested the ability of GPC-expressing cells to form syncytia, we observed that cells expressing GPC_Chimera_ had almost no observable activity at acidic pH compared with cells expressing GPC_MORV_ or GPC_LASV_ (Fig 4E). Overall, the grafted sequence motif is compatible with the primed conformation of GP1 but is poorly compatible with other regions of the spike (likely GP2) when it adopts the native pre-fusion conformation. Thus, we were unable to evaluate the effect of a LAMP1 gain-of-function mutation on cell entry of MORV using the current GPC_Chimera_. Though not informative regarding the effect of introducing LAMP1 binding function into a virus previously lacking it, the results of grafting in the context of the complete spike complex are nevertheless informative because they identify regions of GPC that are important for assembly of the native trimeric spike.

## Discussion

Previous studies have shown that LAMP1 is dispensable for cell entry by LCMV [1, 17]. Here we extended this notion and demonstrated that GP1_LCMV_ does not interact with LAMP1 at acidic pH like GP1_LASV_ (Fig 1A). A recent structure of the LCMV glycoprotein in a pre-fusion conformation [17] revealed a very different architecture for GP1_LCMV_ compared with the LAMP1-compatible conformation observed for GP1_LASV_ [13]. It was tempting to speculate that the GP1_LASV_ structure represents a pH-induced “primed” conformation and that GP1_LCMV_ could also adopt such conformation that might be important for the virus regardless of the use of LAMP1. However, an alternative interpretation of the available structural data would be that the two viruses have fundamental structural differences that explain the differences in ability to bind LAMP1. Thus, a critical question was whether other OW mammarenaviruses can adopt a similar “primed” conformation as seen for GP1_LASV_. The crystal structure of GP1_MORV_ revealed that indeed it adopts this “primed” conformation as GP1_LASV_ (Fig 2A). Therefore, “priming” is common to other OW mammarenaviruses, and inability to bind LAMP1 could not be explained by fundamental structural differences. Instead, side-chain compositional differences and local backbone conformational changes must dictate the ability to bind LAMP1.

We analyzed the various local structural differences between GP1_LASV_ and GP1_MORV_ (Figs 2A, 2C, 3A, 3C & 3D) and used them to guide the mapping of the LAMP1 binding site (Figs 2D, 2E & 3B). We found that the binding of LAMP1 by GP1_LASV_ could be easily disrupted by individual point mutations in a set of multiple residues. The identified residues cluster in a continuous patch on the surface of GP1_LASV_ near the histidine triad, forming a logical footprint for LAMP1 (Fig 3E). This cluster of residues is necessary and sufficient for binding, as grafting it into GP1_MORV_ allowed binding to LAMP1 (Fig 4B & 4C). A central part of the identified LAMP1 binding site consists of strand β5 of GP1_LASV_, which is highly variable in OW mammarenaviruses (Fig 1F). No other OW mammarenavirus seems to have the required residue signature compatible with human LAMP1 (Fig 1F). Even Mopeia virus, which is the closest virus genetically to LASV (Fig 1C), lacks a LAMP1 compatible sequence. Examining representative LASV strains, however, reveals that they all share this unique residue signature (Fig 4F). We thus can conclude based on sequence analyses that LASV but no other OW mammarenaviruses can utilize human LAMP1.

An interesting related question is whether members of this family can utilize LAMP1 orthologs of their host species. It was previously shown that an N-linked glycan at position 76 of human LAMP1 is a critical determinant for binding of LASV, since chicken LAMP1 becomes a functional receptor for LASV when mutated to bear this glycan [1]. Thus, other putative determinants on the surface of LAMP1 that LASV requires in addition to the N-linked glycan at Asn76 must be evolutionary conserved. Considering the large evolutionary distance between humans and chickens, it is likely that the binding surface on LAMP1 would be further conserved in rodent orthologs that are evolutionary much closer to humans than chickens. Taken together, the apparent inability of OW mammarenaviruses to bind human LAMP1 strongly implies that they are also unable to utilize rodent-host orthologs of LAMP1.

Our previous work has shown that LAMP1 triggering greatly enhanced the efficiency of GPC_LASV_-mediated cell entry [14]. We proposed that binding to LAMP1 helps to insure coordinated triggering of multiple spikes at an optimal distance from the membrane [14]. For such coordinated triggering to work, the binding and the subsequent triggering must be fast processes. The mapping of the LAMP1 binding site provides additional support for this idea; based on the available low-resolution EM reconstruction of the LASV spike complex at pH 5.0 and the predicted orientation of GP1 within the complex [11], the three LAMP1 binding sites cluster near the 3-fold symmetry axis of the spike (Fig 3F). If the stoichiometry of LAMP1 binding is three LAMP1 molecules per viral spike complex trimer, the positioning of the binding sites implies that three globular LAMP1 domains cannot bind simultaneously without steric hindrance. Therefore, either a non-globular region of LAMP1 interacts with its binding site in GPC with a 3:3 stoichiometry, or else the stoichiometry is one LAMP1 per GPC trimer (S4 Fig). If the latter is the case then a single LAMP1 binding event might be sufficient for triggering. It should be noted that three binding sites rotated 120 degrees with respect to one another ensures that at least one binding site is favorably posed for interaction when a spike complex approaches a LAMP1 molecule, effectively increasing the on-rate for binding. Such a unique triggering mechanism that enhances the cell-entry efficiency of LASV might be a contributing factor for its pathogenicity, a possibility that will need to be addressed in the future.

Notably, in the EM study by Li S. *et al*. [11], the authors noticed extra densities near the cervixes of the trimer when the structure was reconstructed in the presence of LAMP1 distal domains. These densities were attributed to LAMP1 distal domains, suggesting a stoichiometry of 3 LAMP1 distal-domains per trimer [11]. Our mapping however does not agree with this observation. Analyzing the extra densities reveals that they are likely to be too small to accommodate the expected distal domain of LAMP1, raising the possibility that they were wrongly attributed to LAMP1. Further supporting this notion is the fact that Li S. *et al*. used a LAMP1 distal domain that was produced in the presence of kifunensin [11], an inhibitor that prevents the maturation of N-linked glycans. However, it was shown before that sialylation is critical for making LAMP1 a viable receptor for LASV [1]. This is further corroborated by our observation that GP1_LASV_ fails to bind LAMP1 that was produced in the presence of kifunensin (S3 Fig). Thus, in the absence of fully matured sialylated glycans, a GPC_LASV_/LAMP1 complex is not likely to form.

### Data availability statement

The coordinate file and structure factors of GP1_MORV_ were deposited to the protein data bank and are available under accession code 5NFF.

## Materials and methods

### Expression and purification of recombinant proteins

To express and purify GP1_MORV_ we used the same methodologies as for GP1_LASV_ [13]. Briefly, GP1_MORV_ was expressed as a secreted protein using the baculovirus system in Tni (Trichoplusia ni) cells (Expression Systems). Media were collected and buffer exchanged to TBS (20 mM Tris-HCl pH 8.0, 150 mM sodium chloride) using a tangential flow filtration system (Millipore). Protein was captured using a HiTrap IMAC FF Ni^+2^ (GE Healthcare) affinity column followed by size exclusion chromatography purification with a superdex 75 10/300 column (GE Healthcare). For pull-down assays and SPR analyses, Fc-fused GP1s were expressed in monolayer HEK293T cells (ATCC), and LAMP1 distal domain fused to Fc was expressed in HEK293 cells adapted to suspension (Expression Systems). Transfections were done using linear polyethylenimine (PEI) (25 kDa; Polysciences). Media were collected after 5 days of incubation and supplemented with 0.02% (wt/vol) sodium azide and PMSF.

### Crystallization, data collection, and structure determination

Screening for initial crystallization conditions was done with an 8 mg/ml stock of GP1_MORV_ using a Mosquito crystallization robot (TTP Labs). Initial hits were identified using the PEGRx HT (Hampton) screen and were optimized manually. Crystals of GP1_MORV_ were obtained at 20°C using sitting drop vapor diffusion in 26% PEG 8000, 100 mM sodium citrate pH 4.0, and 0.01% octylphenoxypolyethoxyethanol (IGEPAL). Crystals were then successively cryo-protected using 10% and 25% glycerol in reservoir solution. Details regarding data collection, structure solution and refinement are given in the Supplemental Information.

### Pull-down assays

Media containing GP1-Fc were incubated with Protein A beads (Santa Cruz) for 1 hour at 4°C and washed 3 times with NETI buffer (50 mM Tris-HCl, 150 mM sodium chloride, 1 mM EDTA, 0.5% (vol/vol) IGEPAL). HEK293T cell extracts were prepared in NETI, adjusted to pH 5.0, and incubated for 1 hour at 4°C with the GP1-Fc beads. Proteins were eluted using TBS buffer and precipitated using acetone at -20°C. Pellets were recovered in sample buffer for SDS-PAGE and immunoblot analysis. Anti-LAMP1 antibody (CD107a) was obtained from Millipore and anti-human IgG from Abcam.

### Surface plasmon resonance measurements

The binding of WT GP1_LASV_-Fc, GP1_MORV_-Fc, and their mutants to LAMP1_distal_-Fc was compared using a Biacore T200 instrument (GE Healthcare). Fusion proteins were batch purified using protein A beads in spin columns, and proteins were eluted in 50 mM sodium citrate pH 3, 0.25 M sodium chloride, 0.005% (v/v) tween and 0.02% sodium azide. Purified LAMP1_distal_-Fc was immobilized at coupling density of 1500 response units (RU) on a CM5 sensor chip (GE Healthcare) using primary amine coupling chemistry. One of the four flow cells on the sensor chip was mock-coupled using buffer to serve as a blank. Experiments were performed at 25°C in 50 mM sodium citrate, pH 5.0, 0.25 M sodium chloride, 0.005% (v/v) tween and 0.02% sodium azide. Sensor chip was regenerated using TBS buffer. GP1_LASV_-Fc WT, GP1_MORV_-Fc and their mutated variants were injected at a flow rate of 60 μL/min.

### Structural analysis and electrostatic potential calculations

Partial charges were assigned using PDB2PQR at pH 5.0 [18], and electrostatic potentials were calculated using APBS tools [19] as implemented in the PyMOL Molecular Graphics System, Version 1.8 Schrödinger, LLC. Structural analyses and image generation were done using PyMol.

### Phylogeny analysis

For phylogenetic analysis we used Phylogeny.fr [20]. The diagram was constructed based on GPC sequences obtained from UniProt: Lassa (P08669), Ippy (Q27YE4), Mobala (Q2A069), Dandenong (B1NX58), Mopeia (Q5S586), Morogoro (C6ZK00), Menekre (F1AM21), Gbagroube (F1AM06), Luna (K0IVJ1), LCMV (P09991), and Lujo (C5ILC1) viruses.

### Cell fusion assay

HEK293T cells were seeded in a 24-well plate pre-coated with poly-L-lysine (Sigma). Seeded cells were transfected 24 hours later with 0.2 μg DNA of the indicated GPC constructs using PEI. Twenty-four hours later, cells were rinsed once with HEK293T FM supplemented with 20 mM MES (Acros Organics) and titrated to pH 5.0 or 4.5. Cells were then incubated with the same medium for 10 min, followed by wash and incubation with HEK293T FM for 2 h at 37°C. Following incubation, cells were fixed in 3.7% formaldehyde solution (J.T. Baker) in phosphate buffered saline (PBS) (Biological Industries) for 10 min. Phase images of syncytia were taken at x10 magnification using a phase microscope, and their boundaries were automatically selected using the versatile wand tool of ImageJ.

## Supporting information

S1 Fig Comparison of crystal structure vs. homology model. We used Swiss Model [23] to generate a homology model of GP1_MORV_ based on the crystal structure of GP1_LASV_ (PDB 4ZJF). (A) Superimposition of GP1_LASV_ (grey) and the homology model of GP1_MORV_ (magenta) showing the L7 loop region. The Cα positions and the side-chain orientations are following the GP1_LASV_ template. (B) Superimposition of the homology model of GP1_MORV_ (magenta) and the crystal structure of GP1_MORV_ (blue) showing the L7 loop region. True deviations in backbone conformation are noticeable.
(PDF)

S2 Fig Small variations at the protein core that affect L7 conformation. Leu188 in GP1_LASV_ (right image) is in close proximity to Tyr200. Met187 in GP1_MORV_ (left image), points toward the L7 loop (with its Cε) and may restrict the conformation or position of Tyr200 when Leu188 in GP1_LASV_ is mutated to methionine.
(PDF)

S3 Fig LAMP1 from kifunensine-treated cells cannot bind GP1_LASV_. **(**A) SDS-PAGE with Coomassie staining analysis of protein-A purified distal domain of LAMP1 fused to Fc that was expressed in HEK293 cells with or without Kifunensine treatment. (B) SPR sensogram of GP1_LASV_-Fc at 500 nM that was injected over immobilized distal LAMP1-Fc that was derived from native (blue) or Kifunensine-treated (orange) HEK293 cells.
(PDF)

S4 Fig Binding sites that cluster close to symmetry axis give rise to steric constrains. Schematic diagrams show top views of trimeric spikes with putative binding sites represented as purple circles. Globular binders (hexagons) can bind in a stoichiometric ratio if the binding sites are far enough from the symmetry axis as in ‘A’ but would clash each other if the binding sites cluster near the symmetry axis as in ‘B’, giving rise to sub-stoichiometric binding. To get a 3:3 stoichiometry when the binding sites cluster near the symmetry axis the binder need to have a narrow elongated shape as in ‘C’.
(PDF)
