# Supplementary figures and images for: Mapping of the Lassa virus LAMP1 binding site reveals unique determinants not shared by other old world arenaviruses
Source: PLoS Pathog. 2017 Apr 27;13(4):e1006337. doi: 10.1371/journal.ppat.1006337 (PMC5423696; doi:10.1371/journal.ppat.1006337)

A

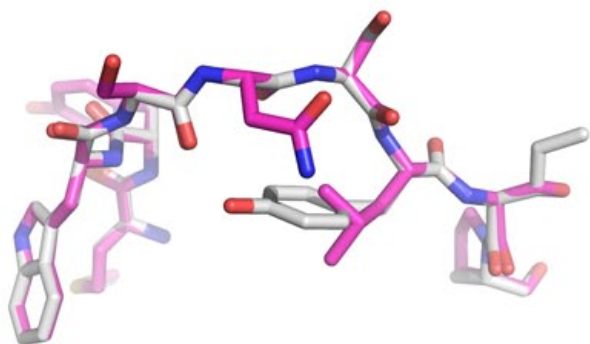

GP1<sub>LASV</sub>

GP1<sub>MORV</sub> - Homology model

B

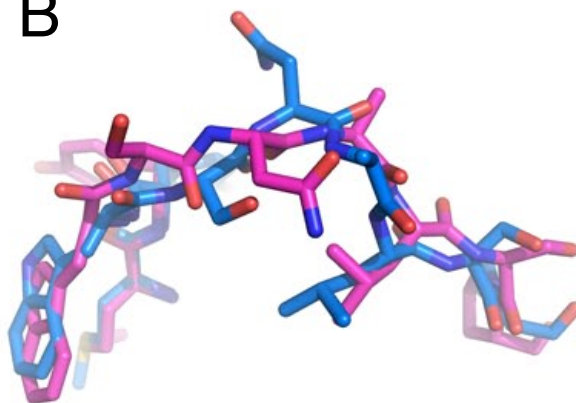

GP1<sub>MORV</sub>

GP1<sub>MORV</sub> - Homology model

Supplement: S1 Fig — We used Swiss Model [23] to generate a homology model of GP1MORV based on the crystal structure of GP1LASV (PDB 4ZJF). (A) Superimposition of GP1LASV (grey) and the homology model of GP1MORV (magenta) showing the L7 loop region. The Cα positions and the side-chain orientations are following the GP1LASV template. (B) Superimposition of the homology model of GP1MORV (magenta) and the crystal structure of GP1MORV (blue) showing the L7 loop region. True deviations in backbone conformation are noticeable. (PDF) [file ppat.1006337.s001.pdf]

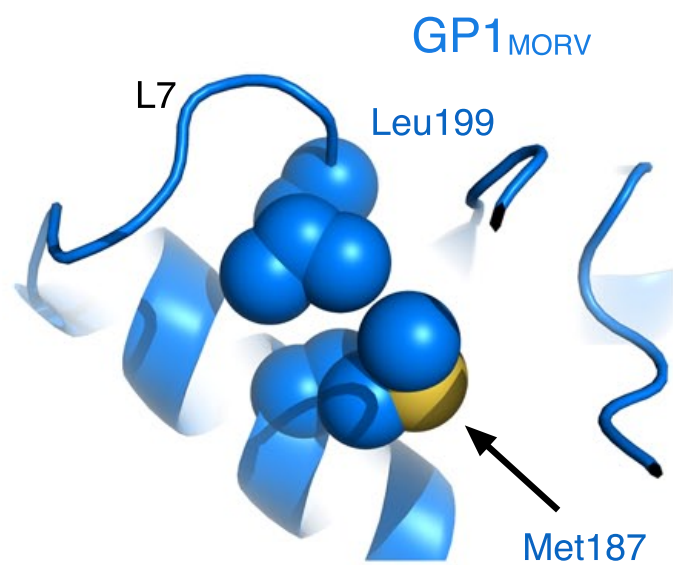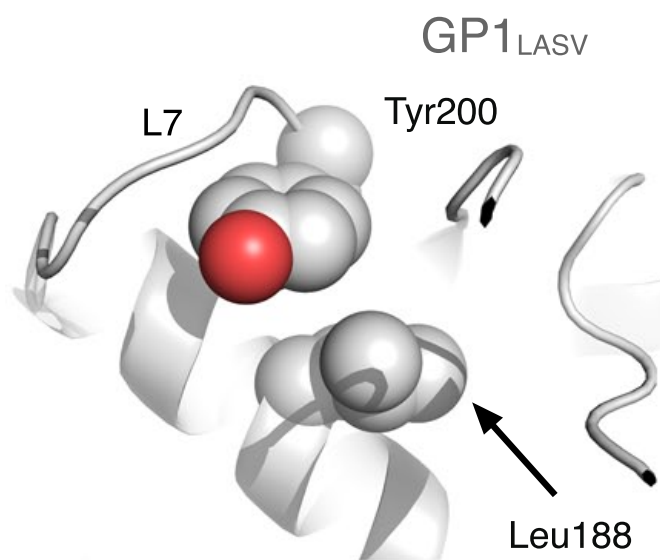

Supplement: S2 Fig — Leu188 in GP1LASV (right image) is in close proximity to Tyr200. Met187 in GP1MORV (left image), points toward the L7 loop (with its Cε) and may restrict the conformation or position of Tyr200 when Leu188 in GP1LASV is mutated to methionine. (PDF) [file ppat.1006337.s002.pdf]

**A**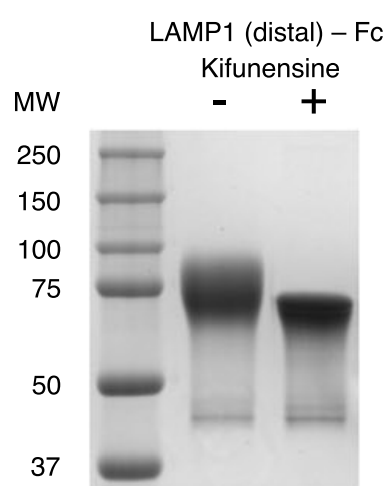**B**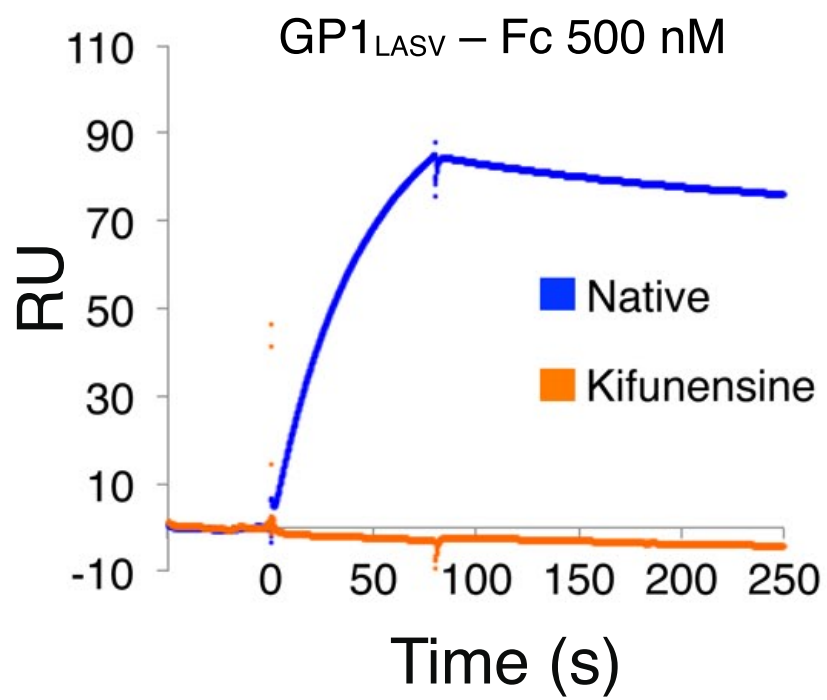

Supplement: S3 Fig — (A) SDS-PAGE with Coomassie staining analysis of protein-A purified distal domain of LAMP1 fused to Fc that was expressed in HEK293 cells with or without Kifunensine treatment. (B) SPR sensogram of GP1LASV-Fc at 500 nM that was injected over immobilized distal LAMP1-Fc that was derived from native (blue) or Kifunensine-treated (orange) HEK293 cells. (PDF) [file ppat.1006337.s003.pdf]

A

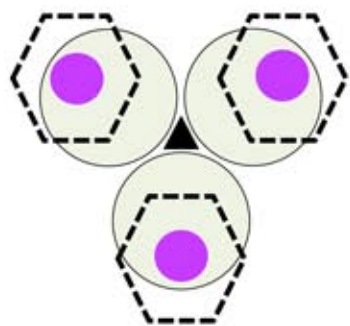

B

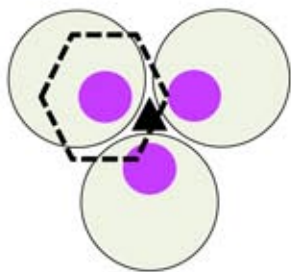

C

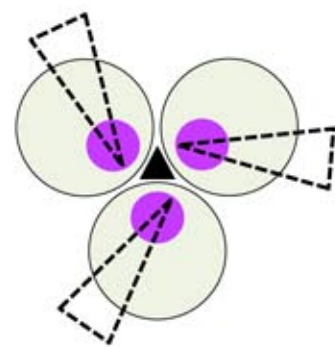

Supplement: S4 Fig — Schematic diagrams show top views of trimeric spikes with putative binding sites represented as purple circles. Globular binders (hexagons) can bind in a stoichiometric ratio if the binding sites are far enough from the symmetry axis as in ‘A’ but would clash each other if the binding sites cluster near the symmetry axis as in ‘B’, giving rise to sub-stoichiometric binding. To get a 3:3 stoichiometry when the binding sites cluster near the symmetry axis the binder need to have a narrow elongated shape as in ‘C’. (PDF) [file ppat.1006337.s004.pdf]
